# Therapeutic targeting of the inflammasome in myeloid malignancies

**DOI:** 10.1038/s41408-021-00547-8

**Published:** 2021-09-14

**Authors:** Samarpana Chakraborty, Lauren C. Shapiro, Sofia de Oliveira, Bianca Rivera-Pena, Amit Verma, Aditi Shastri

**Affiliations:** 1 Division of Hemato-Oncology, Department of Oncology, Albert Einstein College of Medicine, Bronx, NY USA; 2 Department of Developmental and Molecular Biology, Albert Einstein College of Medicine, Bronx, NY 10461 USA; 3 Montefiore Medical Center, Bronx, NY 10461 USA; 4 Department of Medicine (Hepatology), Albert Einstein College of Medicine, Bronx, NY 10461 USA

**Keywords:** Myelopoiesis, Haematological cancer

## Abstract

Even though genetic perturbations and mutations are important for the development of myeloid malignancies, the effects of an inflammatory microenvironment are a critical modulator of carcinogenesis. Activation of the innate immune system through various ligands and signaling pathways is an important driver of myelodysplastic syndromes (MDS) and acute myeloid leukemia (AML). The DAMPs, or alarmins, which activate the inflammasome pathway via the TLR4/NLR signaling cascade causes the lytic cell death of hematopoietic stem and progenitor cells (HSPCs), ineffective hematopoiesis, and β-catenin-induced proliferation of cancer cells, leading to the development of MDS/AML phenotype. It is also associated with other myeloid malignancies and involved in the pathogenesis of associated cytopenias. Ongoing research suggests the interplay of inflammasome mediators with immune modulators and transcription factors to have a significant role in the development of myeloid diseases, and possibly therapy resistance. This review discusses the role and importance of inflammasomes and immune pathways in myeloid malignancies, particularly MDS/AML, to better understand the disease pathophysiology and decipher the scope of therapeutic interventions.

## Introduction

Hematopoiesis is the process of blood cell formation that occurs during embryonic development and across adulthood. It is a complex multifactorial process that operates in response to stimuli from cytokines and transcription factors under stringent regulatory checks [1, 2]. However, perturbations owing to genetic or acquired events that affect any step of the pathway can trigger the development of hematologic disorders, including cancers [3–5]. In particular, uncontrolled proliferation and expansion of abnormal myeloid progenitor cells lead to the development of myeloid malignancies including myelodysplastic syndromes (MDS), myeloproliferative neoplasms (MPNs), and acute myeloid leukemia (AML). Depending on factors such as the subtype of a proliferative cell, response to therapeutics, the necessity for transplantation, etc., patients are categorized into different groups of severity, with AML being the most aggressive with poor long-term survival outcomes. A clinical subtype of AML, secondary AML (sAML), defined as AML occurring after an antecedent myeloid disease, has previously been associated with inferior outcomes compared with de novo AML [6, 7].

Chromosomal abnormalities and gene mutations are known to have strong associations with myeloid malignancies. One-third of AML patients harbor mutations in the FLT3 gene. Despite the nature of mutation-internal tandem duplication (FLT3-ITD) or point mutation (FLT3-TKD), both causes a constitutive activation of tyrosine kinase, leading to proliferation and survival of AML. Patients with FLT3 mutations show poor prognosis, increased risk of relapse, and lower OS [8, 9]. Despite the recent FDA approval of several active agents in myeloid malignancies, including in high-risk MDS and AML, patients with these aggressive diseases eventually relapse unless bridged to an allogeneic stem cell transplant. There is a large unmet need for the development of newer therapeutic agents and rational combinations of these drugs.

Enhanced expression of proinflammatory cytokines such as TNF-α, IL-6, TGF-β, IL-8, and IL-1 in bone marrow (BM) of patients are known to be responsible for ineffective hematopoiesis in MDS [10–14]. The role of the innate immune system in driving these inflammatory signals has now been recognized. The innate immune system is thought to act as a bridge in mediating the effect of the mutations in myeloid progenitor cells to the development of the MDS phenotype with the help of accessory effector proteins [15–17]. Recent studies have emphasized the importance of alarmins to trigger BM expansion of hematopoietic inhibitory myeloid-derived suppressor cells (MDSCs) to activate the inflammasome pathway in MDS [18]. This, in turn, activates a caspase-1 dependent, novel, pro-inflammatory, pyroptosis-mediated lytic cell death in the BM, resulting in the death of healthy hematopoietic stem and progenitor cells (HSPCs) [19]. This pathway is now recognized as an important driver for the development of the MDS phenotype.

Multiple reports have extensively discussed the different classes of myeloid malignancies and mechanisms associated with the pathophysiology of these diseases [20]. This review focuses on highlighting the role of immune dysregulation, and in particular, inflammasomes in the pathogenesis of myeloid malignancies, especially MDS/AML, while touching upon the emergence of promising therapeutic interventions from these pathways.

## The 3I paradigm in myeloid malignancies- immune system, inflammation, and inflammasomes

The role of immune players and inflammation in MDS/AML has been widely studied. While increased apoptosis has been implicated to cause ineffective hematopoiesis in MDS, the cytokine profile and cellular milieu suggests an aberrant innate immune activation in MDS. Elevated levels of pro-inflammatory cytokines such as TNF-α and IL-1 in MDS patients are known to cause the cell death of BM progenitor cells [3, 21–24]. Overall, most studies point to the crucial role of the immune system, inflammation, and inflammasomes in the pathogenesis of myeloid malignancies.

## Immune system and inflammation in MDS

The innate immune system is activated through the interactions of pathogen-associated molecular patterns (PAMPS) against exogenous signals and by damage-associated molecular patterns (DAMPS/Alarmins) against endogenous signals, both of which operate via the activation of pattern recognition receptors (PRRs). Of the known PRRs, Toll-like receptors (TLRs) are the most widely studied, having roles in eliciting innate and adaptive immune reaction and shows an increased expression in inflammatory and autoimmune diseases. TLRs and pro-inflammatory cytokines can activate the hematopoietic stem cells (HSCs) directly [16, 17, 25, 26]. In MDS patients, TLRs are over expressed in HSPCs with respect to age-matched control subjects [27, 28]. In particular, TLR4 is seen to be upregulated in HSPCs of MDS patients and correlates with increased apoptosis of BM-mononuclear cells (BM-MNCs) and CD34^+^ cells. Also, TLR1, TLR2, and TLR6 are significantly overexpressed in the BM CD34^+^ cells of MDS subjects with a higher expression of TLR2 in the BM CD34^+^ cells of low-risk MDS subjects that induces an increase in the level of β-arrestin-1 and cell death. Studies have shown restoration of effective erythropoiesis upon transcriptional silencing of TLR2 [19, 28].

TLR signaling involves two main factors—interleukin receptor-associated kinases (IRAK1 and IRAK4), and TNF receptor-associated factor 6 (TRAF6) that leads to NF-κB and MAPK activation [29]. IRAK4 is a serine-threonine kinase that leads to IRAK1 and TRAF6 activation. We and others have shown that IRAK4 is overactivated in MDS and leads to activation of downstream proliferative pathways. IRAK4 can exist in various isoforms. The longer IRAK4 isoform contains the death domain that interacts with Myd88 allowing signaling downstream of TLR activation and is expressed in higher amounts in MDS patients with U2AF1 splicing factor mutations. Interestingly, IRAK4 activation has also been shown to be involved in resistance to FLT3 inhibitors in MDS/AML [30]. There is an activation of the innate immune pathway via IRAK1/4 complexes that contribute to adaptive immune resistance in FLT3 mutant AML cells [30, 31]. Increased TLR9 expression post-FLT3i treatment activates IRAK1/4 signaling. This increased expression of TLR’s, especially TLR9, has been implicated in the activation of innate immune pathways in adaptively resistant FLT3-ITD AML cells [30].

TRAF6 is an adapter protein that possesses nonconventional E3 ubiquitin ligase activity that mediates signaling from several innate immune receptors and is also reported to be overexpressed in MDS patients with (del)5q mutations [32]. Studies have shown that in (del)5q MDS-HSPCs, deletion of miRNA 146a activates TRAF6 [33, 34]. Such an overexpression of TRAF6 caused hematopoietic defects in a mouse model of MDS, suggesting a connection between immune pathway genes with the pathogenesis of MDS.

A recent report published by Muto et al. [35] studied a cohort of MDS patients in which 40% of MDS patients show overexpression of TRAF6 mRNA with deletion or repression of its negative regulators in MDS-HSPCs. The MDS-HSPCs showed an elevated expression of A20, a dual-ubiquitin (DUB) editing protein that activates the noncanonical NF-κB pathway by terminating the activation of canonical NF-κB via the TLR signaling pathway. This shift is believed to sustain the myeloid expansion and provide a selective advantage for disease cell proliferation as compared to WT-HSPCs, which operates on the canonical NF-κB pathway [35].

Another component of the innate immune system that is overactivated in MDS is the IL-8/CXCR2 pathway. IL-8 has been shown to be overexpressed in MDS stem and progenitor cells and acts via an autocrine manner using CXCR2 receptors. Overexpression of the pro-inflammatory cytokine IL-8 and its receptor CXCR2 is well known for promoting tumor growth and survival, and also as a predictor of adverse prognosis in MDS/AML [36, 37]. Targeting the IL-8/CXCR2 axis in MDS/AML patient cohorts have shown promising results, with decreased viability of primary patients’ HSCs without affecting healthy controls [9]. Also, inhibition of IL-8/CXCR2 signaling has been shown to inhibit MDS stem and progenitors [14]. Furthermore, the interleukin 1 receptor accessory protein (IL1RAP) is overactivated in MDS/AML HSPCs and is enriched in high-risk disease with worse prognosis [3]. Recent data shows that IL1RAP can act as a coactivator for FLT3 signaling thus playing a stimulatory role in malignant myeloid expansion [38]. Overall, these studies show that overexpression of genes involved in innate immune pathways is reported in over 50% of MDS patients [32].

## Alarmins and NLRP3 activation in MDS

S100A8/S100A9 are cytosolic DAMPS, also known as alarmins, that activates the immune signaling system via the TLR-4/NLR pathway and play an important role in inflammatory diseases, including hematologic malignancies. In MDS, the MDSCs are recognized as the key effectors in the development of cytopenias and are associated with the cation binding DAMP heterodimer S100A8/S100A9 complex. The binding of this complex to the CD33 receptor on MDSCs causes activation and expansion of MDSCs, leading to secretion of immunosuppressive cytokines such as IL-10 and TGF-β, along with the further secretion of S100A8/S100A9, thereby initiating a vicious cycle of inflammatory cytokine generation and suppression of hematopoiesis [39]. Transgenic mice expressing S100A9 (S100A9Tg) mimic the features of human MDS, which could be reversed by depletion of MDSCs or by using short hairpin RNA-based (shRNA) silencing of the CD33 receptor, thereby inhibiting the TLR signaling cascade [40].

**Fig. 1:**
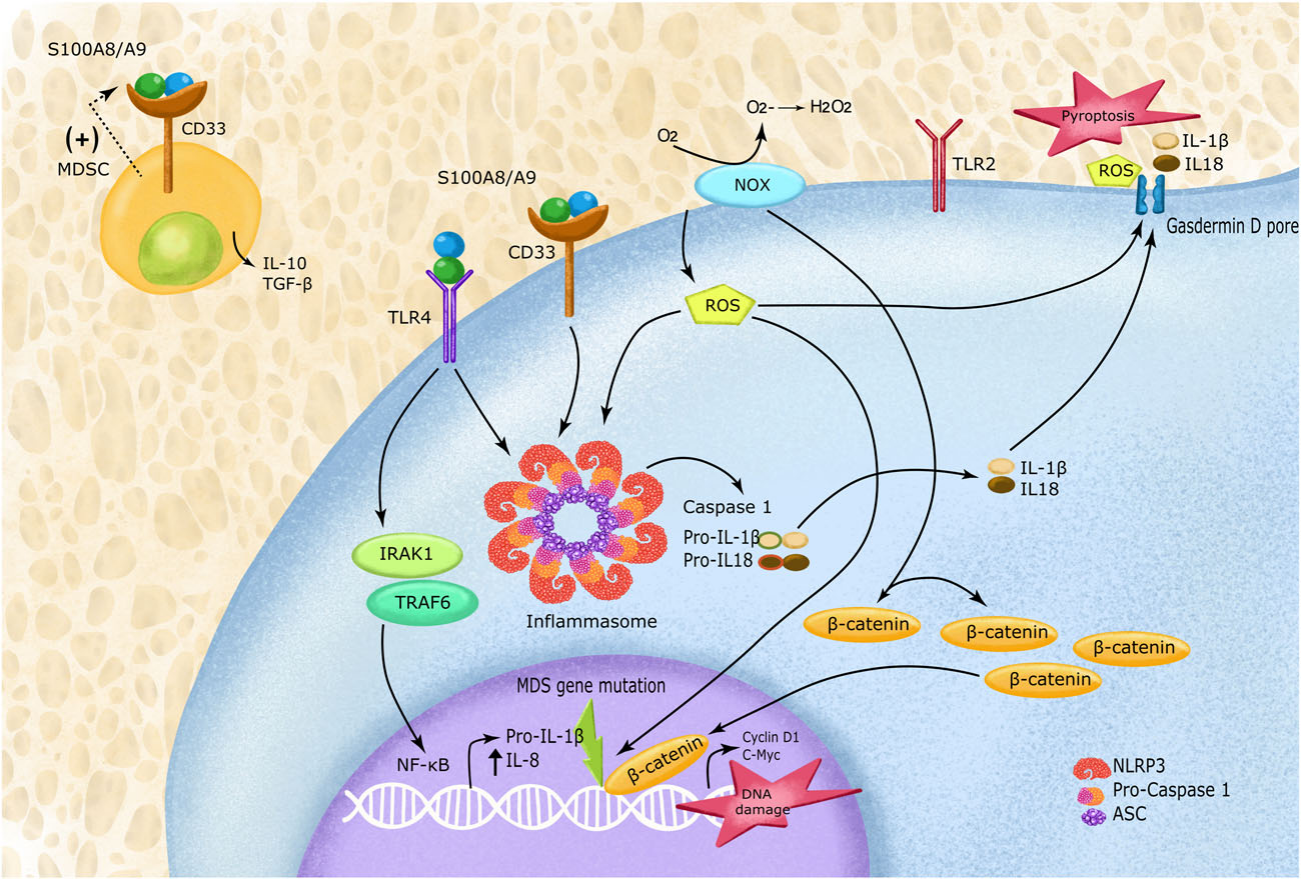
Inflammasome pathway. MDSCs and NLRP3 are activated when DAMPS such as S100A8/S100A9 bind to CD33 and TLR4 receptors, respectively. Activated redox-sensitive NOD-like receptor protein 3 (NLRP3) recruits and causes polymerization of adapter apoptosis-associated speck-like protein containing a caspase-recruitment domain (ASC) proteins to form a complex termed as the inflammasome. This complex serves as a platform for recruitment and autocatalysis of pro-caspase 1 to give rise to active Caspase 1 and thereafter IL-18 and IL-1β from their precursors. Together with heightened ROS level, these cytokines add to the proinflammatory milieu of the cell. This process is followed by the release of proinflammatory cytokines in the cytosol via a lytic cell death called pyroptosis.

The role of alarmins in MDS is twofold: Activating MDSC expansion along with the NOD-like receptor protein 3 (NLRP3) receptor, via the TLR-4 receptor signaling pathway. NLRP3, a cytosolic redox-sensitive sensor, once activated, recruits an apoptosis-associated speck-like protein (ASC) triggering its polymerization and nucleation of large cytoplasmic aggregates to form ASC specks. This complex, termed the inflammasome, facilitates the recruitment and catalytic conversion of pro-Caspase 1 to active Caspase 1, which in turn activates IL-18 and IL-1β. This further enhances the proinflammatory milieu of the cell, activating proliferation via the β-catenin pathway while triggering lytic cell death via a process termed pyroptosis [39]. Caspase 1 activates a pore-forming protein gasdermin D (GSDMD) that oligomerizes and binds to the plasma membrane of myeloid cells to form pores. These pores compromise the membrane integrity, serving as an entry point for cations that release ROS and pro-inflammatory cytokines into the cytosol causing cell swelling and death (Fig. 1). NLRP3 inflammasomes, therefore, leads to multiple faces of MDS pathogenesis—ineffective hematopoiesis, cytopenias, and β-catenin induced proliferation of cancer cells.

The level of S100A9 in plasma is higher in low-risk MDS as compared to high-risk patients [18]. MDS HSPCs and BM plasma reports higher S100A9 protein level as compared to age-matched controls which further increases with hematopoietic lineage progression in MDS BM-MNCs. Furthermore, treatment of normal BM-MNCs with recombinant S100A9 (rhS100A9) is sufficient to induce inflammasome assembly, activation of Caspase 1, and increase in ROS levels. S100A9Tg mouse model shows higher expression of WNT/β-catenin target genes, which is partially reversed by ICTA, an in vivo inhibitor of the NLRP3 inflammasome. Inhibition of inflammasome signaling by either S100A9 neutralization or pharmacological inhibition restores effective hematopoiesis in the S100A9Tg transgenic mouse model [18]. In addition, S100A9 is also known to suppress erythropoietin production, thereby inhibiting erythropoiesis. Lenalidomide treatment is seen to reduce the steady-state generation of S100A9, thereby increasing the levels of Epo and promoting erythropoiesis [41]. This further corroborates the importance of S100A9 in the pathogenesis of MDS.

## Inflammasome activity in AML and other myeloid malignancies

The importance of inflammasomes is also reported in other myeloid malignancies besides MDS. In primary pediatric ALL cells, NLRP3 inflammasome was found to be activated in response to doxorubicin-induced chemotherapy. The p20 subunit of caspase 1 was found to be transcriptionally active in the ALL cells while the NLRP3 expression was found to be modulated by endogenous expression of a cellular DAMP- HMGB1 [42].

A report published by Höckendorf et al., in 2016 suggested a tumor suppressor role of the inflammasome in AML [43]. Failure of inflammasome activation due to loss of RIPK3, a protein kinase, led to the progression from myeloproliferation in FLT3-ITD mutated mice to the development of AML. Contrary to this, subsequent studies reported elevated plasma levels of IL-1β and IL-18 in AML patients as compared to controls [44, 45]. In a panel of 94 cytokines, IL-1β showed the highest effect on the growth of primary AML cells, clustering with GM-CSF and IL-3. In fact, the expression of IL1RAP is now considered a prognostic marker of AML [3] as it is consistently expressed across multiple genetic subtypes of AML and even at the stem cell level. IL1RAP has emerged as an important therapeutic target. It interacts and mediates pro-proliferative effects in AML stem cells through FLT3 kinases. This interaction can be further exploited therapeutically [38]. Interestingly, mRNA expression of NLRP3 and ASC in the BM-MNCs and plasma IL-18 levels show a significant decline in AML patients under complete remission as compared to newly diagnosed ones [44].

A recent article by Hamarsheh et al. [46] shows the activation of an inflammasome pathway in AML patients with KRAS mutation, *Kras*^*G12D*^. A mouse model expressing active *Kras*^*G12D*^ mutation in the hematopoietic system showed myeloproliferation and cytopenia, which can be reversed in *Kras*^*G12D*^ mice with NLRP3 deficiency. The gene expression profile of bone marrow-derived dendritic cells (BMDCs) from either WT or *Kras*^*G12D*^ mice following treatment by tamoxifen have identified NLRP3/caspase1/IL-1β to be a major contributing axis. Oncogenic KRAS is found to produce ROS and thereby activate the inflammasome pathway via RAC1 protein. The findings were confirmed in AML, CMML, and JMML patient samples harboring the KRAS mutation. It is, therefore, possible for inflammasomes to have a diverse role in disease progression, depending on genetic and epigenetic factors.

## The cross-talk between myeloid transcription factors and the inflammasome

Definitive hematopoiesis, which occurs during postembryonic development, engages multipotent HSCs that migrate to the BM and give rise to all blood lineages. HSC maturation involves the differentiation from the blast stage to the diversification of the lineages, giving rise to the lymphoid (T, B, and NK cells), myeloid (granulocytes and macrophages), and erythroid cell lineages (megakaryocytes and erythrocytes) [47, 48].

Two transcription factors, GATA1 and Spi-1 (also known as PU.1), show a cross-inhibitory relationship and are thought to be responsible for the decision of erythroid and myeloid fates [49–51]. Nonetheless, the additional factors and pathways are debated to be responsible for terminal erythroid and myeloid differentiation and their regulation [52–55]. As hematopoietic lineage bias is associated with increased incidence of diseases with prominent inflammatory components, the pathways and players hitherto untapped are believed to hold clinical significance.

**Fig. 2:**
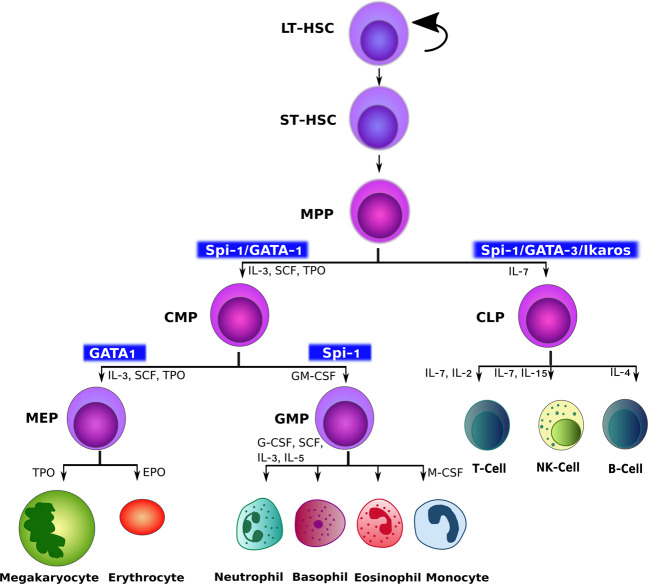
Implications of GATA-1 and Spi-1 in myeloid differentiation of hematopoietic pathway. Hematopoietic stem cells (HSC) evolve from long-term (LT), short term (ST) to multipotent progenitor cells (MPP) from which myeloid and lymphoid cells are formed. At this stage, multiple cytokines along with transcription factors— Spi-1 and GATA1 provide necessary cues for the development of common myeloid progenitor (CMP). However, the next line of myeloid differentiation to megakaryocyte-erythrocyte progenitor (MEP) and granulocyte-monocyte progenitor (GMP) is carried by distinct signaling of GATA1 and Spi-1, respectively. This is followed by the formation of all types of blood cells namely, megakaryocytes (platelets), erythrocytes (RBCs), neutrophils, basophils, eosinophils, and monocytes.

In the lineage progression of HSCs, the presence of Spi-1 and GATA-1 transcription factors are necessary for the formation of common myeloid progenitor cells [52]. Interestingly, in the next step, their levels separately dictate the decision of differentiation into either megakaryocyte-erythrocyte progenitor (MEP) or granulocyte-monocyte progenitor (GMP), as shown in Fig. 2. Tyrkalska et al. [56] suggest that an underlying inflammatory background is responsible for the imbalance in the ratio of GATA-1: Spi-1 and subsequently, the decision of the lineage and its bias. Inflammasomes are thought to be prerequisite for myeloid differentiation that operates in an evolutionarily conserved mechanism by regulating the GATA1: Spi-1 ratio in the cells. In a zebrafish model, inflammasome deficient larvae express higher GATA1 at transcript and protein levels, inhibiting myeloid differentiation and enforcing erythropoiesis. As expected, pharmacological inhibition of the inflammasome rescues anemia and neutrophilic inflammation in the disease model. In mouse HSCs, Caspase 1 inhibitor upregulates GATA1 levels, with a direct increase in megakaryocyte-erythrocyte (MegE) colonies and a decrease in granulocyte-monocyte (GM) colonies. Pharmacological inhibition of the inflammasome in human erythroleukemic K562 cells leads to the suppression of erythroid differentiation with a decline in hemoglobin accumulation and decreased GATA1 levels as compared to DMSO treated control cells. In human HEK293 cells, Caspase 1 directly cleaves GATA1 at residue D300 thereby causing its degradation, indicating the likely reason of GATA1 accumulation on Caspase 1 inhibition. This evolutionarily conserved role of the inflammasome in the regulation of erythroid versus myeloid fate suggests a potential new area of drug development [56].

Innate immune players such as TNF-α and IL-1β are known to upregulate Spi-1 protein in HSC in vitro and in vivo, via activation of inflammatory signaling [56–58]. As these proinflammatory cytokines are the released byproducts of inflammasome formation, it is hypothesized that the mechanism of upregulation of Spi-1 is superseded by GATA1 downregulation. While few studies have addressed the missing links in this area of research, the underlying mechanism of immune regulation, inflammation, and lineage bias in myeloid malignancies are not well understood and is still in its infancy.

## Is inflammasome pathway the converging point for the development of MDS phenotype?

A recent review discussed the impact of genetic abnormalities and their relation to immune and inflammasome signaling in MDS [19]. For multiple genetic events, be it somatic mutations or chromosomal abnormalities, a different player of the immune system comes into action. For example, epigenetic modifications in different genes activate distinct immune signaling cascade. TET2 mutants increase IL-6 production via a decrease in HDAC2 recruitment with a concomitant increase in IL-1β while ASXL1 mutation activates TLR4 signaling and increases NADPH oxidase, and in turn ROS levels [18, 59, 60]. A similar trend can be observed for mutation in spliceosome genes. For example, NF-κB activation in the SF3B1 mutant occurs by downregulation of MAP3K7, but for the SRSF2 mutant, the activation is due to caspase 8 isoform, along with an increase in alarmin levels [18, 61]. Chromosomal abnormalities such as (del)5q mutation causes haploinsufficiency, increasing alarmins and miR-145/146 levels and subsequently activating TRAF6/IRAK1 signaling [62]. All these mutations and diverse effectors that trigger the activation of different immune signaling pathways point towards an interesting observation, a unanimous activation of pyroptosis and the β-catenin signaling pathway. This suggests the possibility of inflammasomes playing a central role in the pathogenesis of MDS/AML and the importance of exploiting this pathway to identify novel therapeutic targets in MDS/AML.

### Therapeutic avenues for inhibiting the myeloid inflammasome

Previous sections of this review have established that innate immune signaling and subsequent inflammasome activation via NLRP3 plays a major role in the proliferation and sustenance of MDS HSPCs. Inflammasome activation has also been linked to several inflammatory diseases, including MDS. Thus, players involved in the inflammasome pathway can work as excellent pharmacological targets against MDS/AML. In this section, we discuss multiple mechanisms by which inflammasome formation and activity can be inhibited, and the early-phase clinical trials showing promise for future clinical practice.

As described before, the alarmins- S100A8/S100A9 bind the CD33 receptor on MDSCs leading to their activation, expansion, and ultimate suppression of hematopoiesis. Several monoclonal antibodies, antibody-drug conjugates (ADC), a bispecific T-cell engager (BiTE), and a trispecific killer engaged molecule (TriKE) against CD33, have been developed in hopes of suppressing MDSC proliferation and renewing hematopoiesis in MDS/AML.

Lintuzumab, a CD33 humanized monoclonal antibody was studied in older patients with untreated AML. However, the randomized Phase IIb trial failed to show improvement in overall survival (OS) in a cohort of 211 patients treated with Lintuzumab in combination with cytarabine as compared to cytarabine alone (4.7 months versus 5.1 months), thereby suspending its further clinical development [63]. A novel radioimmunoconjugate using Lintuzumab, ^225^Ac-lintuzumab, however, has shown early promise in preliminary phase II data in older AML patients unfit for induction chemotherapy who express CD33 on >25% of blasts [64]. ^225^Ac-lintuzumab links Lintuzumab to a short-range, high-energy, cytotoxic alpha-particle emitter (^225^Ac) which uses radiotherapy to elicit single and double-strand DNA breaks in selectively targeted CD33 cells. Preliminary data has been reported on nine patients treated with ^225^Ac-lintuzumab monotherapy showing an overall response rate (ORR) of 56% with two complete remissions with incomplete platelet recovery (CRp) and three complete remissions with incomplete hematologic recovery (CRi) [64]. ^225^Ac-lintuzumab in combination with Venetoclax is also being studied in an early phase I/II trial in relapsed/refractory AML (NCT03867682).

BI 836858, another CD33 monoclonal antibody glycoengineered against CD33 has also not shown much promise in untreated patients with MDS/AML, with only 18% patients achieving a CR/CRi among 28 patients with untreated AML in the phase I/II dose-escalation study of BI 836858 in combination with azacitidine (AZA) as part of the Beat AML dataset [65]. A similar phase I/II study (NCT02240706) using BI 836858 in patients with low or intermediate-1 risk MDS has been terminated as the company decided to stop the clinical development of BI 836858 prematurely for strategic reasons. As per the study results listed on clinicaltrials.gov, it appears this drug was limited by both efficacy and toxicity.

Two CD33 ADC have been developed, vadastuximab talirine and IMGN779, however, all clinical trials of vadastuximab talirine were suspended after the phase III CASCADE trial (NCT02785900) was terminated in 2017 after reporting a higher rate of deaths including fatal infections compared to the control arm despite promising initial phase I data showing an ORR of 70% [66]. IMGN779, however, is an ADC using an indolinobenzodiazepine pseudodimer payload, with encouraging initial phase I results showing manageable tolerability and anti-leukemia activity in patients with relapsed/refractory AML [67].

Although CD33 targeting has not shown much promise in humans thus far, the newest BiTe and TriKE agents are using innovative approaches to target MDSC production. AMV564 is a novel bivalent, bispecific, CD33/CD3 T-cell engager that binds CD33 on target cells and CD3 on T-cells leading to T-cell-directed lysis of CD33^+^ leukemic blasts and MDSCs, as well as T-cell expansion, differentiation, and proliferation [68]. GTB-3550 is a novel CD16/IL-15/CD33 TriKE that induces natural killer cell function by targeting malignant cells as well as the CD33^+^ MDSC, which contribute to tumor-induced immunosuppression. Because CD16 is the most potent activating receptor on NK cells, this single agent may induce a targeted cytolytic anti-CD33 tumor response [69]. Both agents are currently in early phase clinical trials in MDS/AML patients (Table 1).

**Table 1:** Potential therapeutic agents targeting the inflammasome pathway in myeloid malignancies.

Target biomolecule	Therapeutic agent	Feature	Disease targeted	Stage of development	Clinical trial identifier
CD33 receptor	BI 836858	Anti-CD33 monoclonal antibody	MDS AML	Phase 1/2 terminated Phase 1	NCT02240706 NCT03013998
	AMV564	CD33/CD3 BiTE	Int/HR-MDS/AML/advanced solid tumors	Phase 1	NCT03516591 NCT03144245 NCT04128423
	GTB-3550	Trispecific killer engager molecule (TriKE) against CD16/IL-15/CD33	HR-MDS/AML/ mast cell leukemia	Phase 1/2	NCT03214666
	IMGN779	CD33 antibody-drug conjugate	AML	Phase 1 completed	NCT02674763
	Lintuzumab	Anti-CD33 monoclonal antibody	AML	Phase 2 completed	NCT00528333
	^225^Ac-lintuzumab	Radioimmunoconjugate against CD33	AML	Phase 1/2 Phase 1/2	NCT02575963 NCT03867682
	Vadastuximab talirine	CD33 antibody-drug conjugate	AML	Phase 3 terminated	NCT02785900
TLR2	OPN-305	Anti-TLR2 monoclonal antibody	LR-MDS	Phase 2 completed	NCT02363491 NCT03337451
TLR4	Bortezomib	Proteasome inhibitor modulating TLR4 activity	LR/Int-MDS	Phase 2 completed	NCT01891968
NLRP3	MCC950	Blocks NACTH ATPase domain of NLRP3	MDS	In vitro/in-vivo	N/A
	MNS	Inhibits NLRP3 ATPase activity by cysteine modification	MDS	In vitro/in-vivo	N/A
	CY-09	Inhibits NLRP3 ATPase activity to block NLRP3 activation	MDS	In vitro/in-vivo	N/A
	OLT1177		Osteoarthritis, Schnitzler Syndrome	Phase 1/2	NCT01768975NCT03595371
ASC	Ibrutinib	BTK inhibitor that also binds to ASC and inhibits ASC aggregation	CLL HR-MDS AML	Phase 2 Phase 1 Phase 2 terminated	NCT03207555 NCT03359460 NCT02553941 NCT02351037
Caspase 1	VX-765	Peptidomimetic drug that blocks caspase 1 active site	Epilepsy/psoriasis	Phase 2 completed	NCT01048255 NCT00205465
	Parthenolide analog	Plant-based Michael acceptor inhibitor that directly inhibits caspase 1	Contact dermatitis detection	Phase 3 completed	NCT00640614
IL-1β	Canakinumab	IL-1β neutralizing monoclonal antibody	MDS/CML	Phase 1 Phase 2	NCT04810611 NCT04239157
	Rilonacept	Soluble decoy receptor that binds to IL-1β/IL-1α	Cryopyrin-associated periodic syndromes (CAPS)	N/A	N/A
	Anakinra	Antagonist of IL-1R	Cryopyrin-associated periodic syndromes (CAPS)	N/A	N/A
IRAK	CA - 4948	Small molecule inhibitor against IRAK4	MDS	Phase 1/2	NCT04278768
Wnt/β-catenin	CWP232291	Small molecule inhibitor of Wnt/β-catenin	MDS/AML	Phase 1 completed	NCT01398462

Instead of targeting MDSC’s directly through CD33, inhibitors that specifically target TLR signaling and their downstream effectors, IRAK and the NLRP3 inflammasome, may provide another avenue to disrupt pyroptosis-mediated cell death of HSPC’s and β-catenin-induced proliferation of cancer cells. Most promising has been the TLR2 humanized monoclonal antibody Tomaralimab (OPN-305). In a phase II trial using Tomaralimab in heavily pretreated, transfusion-dependent patients with low or intermediate-1 risk MDS after failure with prior hypomethylating agents, 50% (6/12) patients had an overall response in the form of hematologic improvement with 17% (2/12) patients achieving transfusion independence. This therapy was well tolerated without significant toxicities [70]. Another potential way of targeting TLR signaling may be through proteasome inhibition. In an exploratory clinical trial using bortezomib in 15 patients with low-risk MDS, investigators noted that proteosome inhibition with bortezomib modulated TLR signaling. This occurs by decreasing levels of phosphorylated p65, a surrogate for NF-κB activation that leads to a significant p65 downregulation in 54% of patients, which correlated with clinical response, albeit only 20% of patients had evidence of short-lived hematologic improvement [71]. The investigators argued that using a drug with more potent and specific inhibition of the NF- κB pathway could lead to a more significant and long-lasting clinical response. One potentially attractive target is the IRAK4 inhibitor CA-4948, which directly inhibits NF-κB activation through its interaction with MyD88/IRAK1/TRAF6 [72]. A phase I dose-escalation trial using CA-4948 in adult patients with AML or high-risk MDS is currently enrolling and an early report shows the drug to be safe and well tolerated (NCT04278768) [73]. IRAK1/4 is also an attractive immune target in therapy-resistant myeloid malignancies and as previously mentioned, contributes to adaptive immune resistance in FLT3 mutant AML cells that can be therapeutically exploited. A small molecule dual inhibitor of FLT3/IRAK4 can overcome adaptive resistance in FLT3-ITD AML preclinical models by inhibiting compensatory IRAK 1/4 activation and downstream immune activation in FLT3-ITD AML [30]. Furthermore, a combinatory inhibitor targeting the FLT3/IL-8-CXCR2 axis may also serve to overcome the FLT3 resistance in AML.

In addition, a phase I study evaluating the small molecule CWP232291, an inhibitor of Wnt signaling that leads to direct degradation of β-catenin, has shown proof of concept that directly targeting downstream β-catenin cell proliferation maybe another novel mechanism for eradication of early leukemic progenitors. Further exploration as combination therapy is likely required [74].

Sustained levels of IL-1β have also been shown to activate NF-κB and MAPK signaling leading to an increase in IL-6 production. Furthermore, it supports MDSC accumulation as observed by the IL-1 receptor-deficient mouse model that shows inhibition of tumor progression due to delayed MDSC accumulation. Anti-IL-1β targets such as IL-1β neutralizing antibody Canakinumab [75], soluble decoy IL-1 receptor Rilonacept, and recombinant IL-1 receptor antagonist Anakinra [76] have been approved for autoinflammatory diseases. These can also be promising clinical targets against MDS, [77] with two early phase trials using Canakinumab in low-risk MDS currently recruiting (NCT04810611 and NCT04239157). However, targeting IL-1β alone may be insufficient as other cytokines such as IL-18 are also involved in adding to the pro-inflammatory milieu.

As previously mentioned, IL1RAP, a surface molecule that is consistently overexpressed on AML stem cells interacts with FLT3 kinases and is an important therapeutic target. Anti IL1RAP/CD3 bispecific antibodies have demonstrated a high degree of specificity and IL1RAP targeting therapies are now going into first-in-human clinical trials (NCT02842320) in leukemias [78].

Inhibitors that specifically target NLRP3 have not yet made it to the clinic for MDS/AML, but are currently under preclinical development, and have shown promising results with lesser toxicity. The NLRP3 inhibitor MCC950 showed high specificity for both canonical and noncanonical inflammasome pathway activation and significant efficacy at nanomolar concentrations [79, 80]. However, anecdotal reports of hepatotoxicity in patients with rheumatoid arthritis in early phase trials blocked its further development [67]. Other direct inhibitors of NLRP3 such as 3,4-methylenedioxy-β-nitrostyrene (MNS) and CY-09 (an analog of CFTR inhibitor-172 (C172) which inhibits the CFTR channel), specifically inhibits ATP binding of NLRP3 without affecting other NOD-like receptors. Tranilast and OLT1177 have shown promising results in animal models and ex vivo studies and seem to show significant potential as drug targets against NLRP3 related diseases [81–83].

Bruton tyrosine kinase (BTK) also regulates NLRP3 inflammasome activity by direct interaction with ASC and NLRP3. Ibrutinib-a BTK inhibitor prevents the formation of ASC specks and Caspase 1 activation. It was shown to suppress IL-1β maturation and caspase 1 activation in PBMCs of Ibrutinib treated cancer patients and is currently under phase 1 trials in combination with lenalidomide and 5′-Azacytidine for MDS (NCT03359460 and NCT02553941). Unfortunately, a phase II trial of ibrutinib alone or in combination with cytarabine or AZA in AML patients unfit for standard therapy or with relapsed/refractory disease, did not show any clinically relevant anti-leukemia activity [84]. Orally active Caspase 1 inhibitor such as VX-765 [85] and other caspase 1 inhibitors such as soluble analogs of Parthenolide (anti-inflammatory sesquiterpene lactone compound) can also be potential drug targets in hematological lineage bias disorders such as MDS and AML, which have only been studied in epilepsy and dermatologic conditions so far. Interestingly, recent reports have identified GSDMD to be a specific effector protein triggering pyroptosis, making it an attractive target which could have an important future role in immune therapeutic approaches designed to target specifically pyroptosis as an inflammasome activation downstream effect [81, 86].

**Fig. 3:**
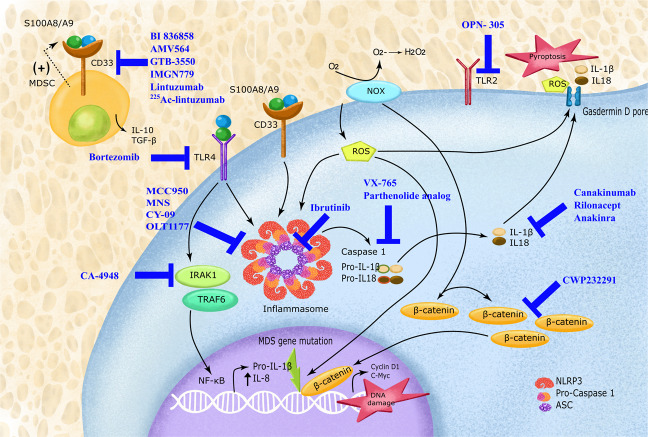
Emerging therapeutic targets from the immune and inflammasome pathway in MDS. The figure depicts the various biomolecules from the inflammasome pathway that are targeted by the novel therapeutic agents in MDS and AML.

Inflammasomes and immune response pathways have opened avenues for several exciting new drug targets for MDS and AML. As inflammasomes appears to play a role in MDS originating from multiple genetic defects, promising outcomes are expected from its drug targets. Table 1 and Fig. 3 shows the potential therapeutic agents targeting the players of the inflammasome pathway in myeloid malignancies.

## Conclusion

Identification of novel pathways that are integral to the myeloid disease pathophysiology can guide the field towards newer targets for therapy. Often, pretransplant candidates with FLT3-ITD mutated AML develop rapid resistance to the FLT3 inhibitors. As pointed out, inflammasome targeting therapies can be explored as combinatorial strategies with FLT3 inhibitors to overcome therapy resistance as ample evidence points towards possible synergistic mechanisms. Underlying genetics and chronic inflammation can activate the innate immune system to trigger the inflammasome pathway, which seems to play a central role in the pathophysiology of myeloid malignancies. While DAMPS such as S100A8/S100A9 are known to activate the inflammasome pathway, the signaling mechanism that increases the DAMP levels in the first place remains unknown. Even though a number of drug targets have been identified from this pathway, it is a relatively new field in the context of myeloid malignancies. We anticipate that understanding the inflammasome pathway, its activators, inhibitors, and effectors will be crucial for further identification of novel and improved therapeutic outcomes against these diseases that currently have a dismal prognosis. With several therapies targeting the inflammasome currently in clinical development, it is our sincere hope that some of them will come to fruition and we will soon have an FDA-approved inflammasome targeting drug in a clinic for our patients with aggressive myeloid malignancies.
